# RNA-Seq and WGCNA Analyses Reveal Key Regulatory Modules and Genes for Salt Tolerance in Cotton

**DOI:** 10.3390/genes15091176

**Published:** 2024-09-07

**Authors:** Bo Pang, Jing Li, Ru Zhang, Ping Luo, Zhengrui Wang, Shunyu Shi, Wenwei Gao, Shengmei Li

**Affiliations:** 1 College of Agriculture, Xinjiang Agricultural University, Urumqi 830052, China; 13894100819@163.com (B.P.); 15739671550@163.com (J.L.); zr13094012400@163.com (R.Z.); luoping987@126.com (P.L.); 15569049878@163.com (Z.W.); 15739283339@163.com (S.S.); 2 College of Biotechnology, Xinjiang Agricultural Vocational and Technical University, Changji 831100, China

**Keywords:** *Gossypium hirsutum*, salt stress, RNA-seq, WGCNA

## Abstract

The problem of soil salinization has seriously hindered agricultural development. Cotton is a pioneering salinity-tolerant crop, so harvesting its key salinity-tolerant genes is important for improving crop salt tolerance. In this study, we analyzed changes in the transcriptome expression profiles of the salt-tolerant cultivar Lu Mian 28 (LM) and the salt-sensitive cultivar Zhong Mian Suo 12 (ZMS) after applying salt stress, and we constructed weighted gene co-expression networks (WGCNA). The results indicated that photosynthesis, amino acid biosynthesis, membrane lipid remodeling, autophagy, and ROS scavenging are key pathways in the salt stress response. Plant–pathogen interactions, plant hormone signal transduction, the mitogen-activated protein kinase (MAPK) signaling pathway, and carotenoid biosynthesis are the regulatory networks associated with these metabolic pathways that confer cotton salt tolerance. The gene-weighted co-expression network was used to screen four modules closely related to traits, identifying 114 transcription factors, including WRKYs, ERFs, NACs, bHLHs, bZIPs, and MYBs, and 11 hub genes. This study provides a reference for acquiring salt-tolerant cotton and abundant genetic resources for molecular breeding.

## 1. Introduction

Soil salinization is an escalating global concern, with detrimental effects on plant development that lead to reductions in crop productivity. It is estimated that 20% of irrigated land was affected by soil salinization a decade ago, and without intervention, saline soils could expand to more than 50% of the total global irrigated area by 2050 [1,2]. Apart from the natural soil enrichment process, intensified agricultural irrigation practices and the negative influence of climate change have exacerbated this issue [3]. In turn, the economic fallout from crop failures due to heightened soil salinity has provoked shifts in migration patterns among farmers in coastal agrarian regions like Bangladesh [4]. Consequently, there is a pressing need to enhance crop tolerance to saline–alkaline conditions to address this escalating crisis. Excessive salinity not only restricts a plant’s ability to absorb water and nutrients [5], but also triggers a cascade of stress responses, including responses to osmotic pressure, ionic imbalance, and oxidative stress [6].

As we usher in the post-genomic age, the swift progression of transcriptomics technology has revealed many correlations between genomes, phenotypes, tissue specifics, developmental phases, and environmental influences [7]. By leveraging transcriptomes’ temporal and spatial variability, this innovative technology can be used to identify genes that govern plant growth, development, and stress resilience, accelerating the process of crop variety enhancement [8]. Segura et al. studied the transcriptomic shifts at the stem apex of wild-type and male ethylene-insensitive cucumber mutants during the female flowering transition. A total of 1160 distinctive differential expression genes DEGs were identified in the wild type’s female blooming phase, 284 of which were influenced by the etr1b mutation, which impairs ethylene sensitivity. These genes belong to 34 distinct transcription factor families, including NAC, ERF, bHLH, bZIP, MYB, and C2H2/CH3, and have been shown to play roles in modulating female flowering, either in an ethylene-dependent or non-ethylene-dependent manner [9,10]. Aleen et al. studied drought resilience through a comparative transcriptomic analysis of drought-tolerant and -sensitive soybean root systems, finding that DEGs associated with water and growth hormone transportation, cellular membrane and wall functions, antioxidative activity, catalytic processes, secondary metabolism, signaling pathways, and transcriptional regulation are localized in a key QTL hotspot region on Chr.08 for drought response. Within this hotspot, eight single-nucleotide polymorphisms (SNPs) were identified as potential candidate genes governing drought tolerance [11]. Liu et al. identified twenty-two, nine, and six distinct salinity-sensitive genes within the phytohormone signaling, MAPK signaling, and abscisic acid (ABA) signaling cascades, respectively. Through single-cell RNA-seq analysis, they predicted a pivotal function for the AP2/ERF transcription factor family in cotton root-tip protoplasts subjected to salt stress [12,13]. In a separate study, a transcriptomic sequencing investigation of the salt-resistant cotton cultivar Tong Yan No. 1 revealed significant changes in metabolic pathways in reaction to salt stress; these pathways included glycerolipid metabolism, sesquiterpene and triterpene synthesis, flavonoid production, and phytohormone signaling. This study also validated the functionality of two candidate genes, *Gh_D11G0978* and *Gh_D10G0907*, through the virus-induced gene silencing (VIGS) technique [14].

The research on salt tolerance in cotton at the transcriptomic level has predominantly centered on comparatively sequencing individual cultivars or singular temporal phases [14,15]. However, the dynamic regulatory mechanisms underlying salt stress defense across diverse cultivars remain largely unexplored. In this study, we contrasted the transcriptome profiles of various *G. hirsutum* genotypes under salt stress conditions, aiming to discern the shared and distinct defense reactions between salt-tolerant and -sensitive types. This research reveals the key regulatory pathways integral to cotton’s salt tolerance response and provides genetic information useful for molecular breeding.

## 2. Materials and Methods

### 2.1. Material Planting and Sample Preparation

Cotton seeds were delinted, washed, and soaked in distilled water for 24 h to promote seed germination, and the dried and broken seeds were removed. Nutrient soil was mixed with vermiculite in a 1:1 ratio, filled to 1/3 height in a 10 × 10 nutritive bowl, and compacted, and full seeds were inserted into the soil with tweezers whose tips faced downward. Five seeds were placed evenly in each bowl. Then, we covered the seeds with nutrient soil up to 3/4 of the height of the seedling bowl, poured 1 L of water into the tray, and wrapped the bowl tightly in a black plastic bag to moisturize the soil. We removed the plastic film when the seedlings emerged evenly, pulled out the weak seedlings, and left 3 cotton plants in each bowl. When the cotton plants reached the third-leaf stage at around three weeks of age, we delicately cleansed the roots, ensured their integrity, and placed them in a conical flask. The seedlings were immersed in Hoagland nutrient solution for two days. After two days, the Hoagland nutrient solution was extracted, and the experimental group was treated with 350 mM of NaCl. The control group was given an equal amount of distilled water [16,17]. The leaves were removed at 0 h, 2 h, 5 h, 12 h, 24 h, 48 h, and 72 h after treatment; wrapped in tin foil; and placed in freezing tubes. These were immediately placed in liquid nitrogen for preservation, with three replicates for each group. The leaves were then submitted to Qingdao Biomarker Biotechnology Co (Qingdao, China). for library construction and sequencing.

### 2.2. Library Construction and Sequencing

Total plant RNA was extracted using the RNA prep Pure Plant Kit (Tiangen, Beijing, China), and RNA concentration and purity were measured using a NanoDrop 2000 (Thermo, Wilmington, DE, USA). Sequencing libraries were generated using the Hieff NGS Ultima Dual-mode mRNA Library Prep Kit for Illumina (Yeasen, Shanghai, China) according to the instructions provided by the manufacturer. The library fragments were purified using AMPure XP magnetic beads (Beckman Coulter, Beverly, MA, USA). Then, 3 μL of USER Enzyme (NEB, Ipswich, MA, USB) was added, and the sample was incubated at 37 °C for 15 min. The reaction was performed at 95 °C for 5 min before conducting PCR analysis to purify the PCR products (AMPure XP system) and assess library quality using an Agilent Bioanalyzer 2100 system (Agilent, Santa clara, CA, USA). The libraries were sequenced using the Illumina NovaSeq. (Illumina, San Diego, CA, USA) platform to generate a 150 bp double-terminal sequence.

### 2.3. Sequencing Data Quality Control and Analysis

Clean data (clean reads) were obtained by removing reads containing adapters, reads containing ploy-N, and low-quality reads from the raw data. All the downstream analyses were based on clean data with high quality. These clean reads were then mapped to the reference genome sequence, TM-1 (https://www.cottongen.org/species/Gossypium_hirsutum/HAU-AD1_genome_v1.0_v1.1, accessed on 29 March 2023), and annotated based on the reference genome. Hisat2 tools software (Hisat2_v2.2.1) was used to map the reads with the reference genome [18]. The StringTie Reference Annotation Based Transcript (RABT) assembly method was used to construct and identify both known and novel transcripts from the Hisat2 alignment results [19]. Gene functions were annotated via Blastp using the following database: Nr (NCBI non-redundant protein sequences), KOG (Clusters of Orthologous Groups of proteins), KO (KEGG Ortholog database), and GO (Gene Ontology) [20,21,22,23]. Gene expression levels were estimated in terms of fragments per kilobase of transcript per million fragments mapped (FPKM) [24].

### 2.4. Differentially Expressed Gene Screening and Analysis

Differential expression analysis of cotton cultivars and treatments was performed using DESeq2 [25], and genes with an FDR < 0.05 and Fold Changes ≥ 2 were designated DEGs.

### 2.5. Weighted Gene Co-Expression Network Construction

The gene co-expression network was constructed using the “WGCNA” R package (https://cran.r-project.org/web/packages/WGCNA/index.html, accessed on 1 March 2024). The Pearson correlation coefficients between genes were calculated based on the expression of genes in different samples; the correlation coefficients were converted into a neighbor-joining matrix through a weighting function; and a soft threshold (β) was determined for the neighbor-joining function to make the gene expression matrix conform to the scale-free network. Gene modules were visualized using Cytoscape (Cytoscape_v3.9.1). The hub gene screening parameter values were set as follows: weight > 0.35 and degree > 5 [26].

### 2.6. qRT-PCR Validation

Based on the RNA-seq results, six DEGs were randomly selected to verify the accuracy of the sequencing results. Leaf RNA was taken from 3 replicate samples from 2 species under 7 conditions for qRT-PCR amplification after inverse transcription. Specific primers were designed according to DEG annotation using the NCBI’s primer-designing tool (https://www.ncbi.nlm.nih.gov/tools/primer-blast/, accessed on 14 May 2024). QRT-PCR amplification was performed using the Applied Biosystems 7500 Fast real-time fluorescence Quantitative PCR Instrument System (Thermo, Wilmington, DE, USA), and relative gene expression levels were analyzed using GraphPad Prism 9.5.0 [27].

## 3. Results

### 3.1. Transcriptome Sequencing and Assembly

**Figure 1:**
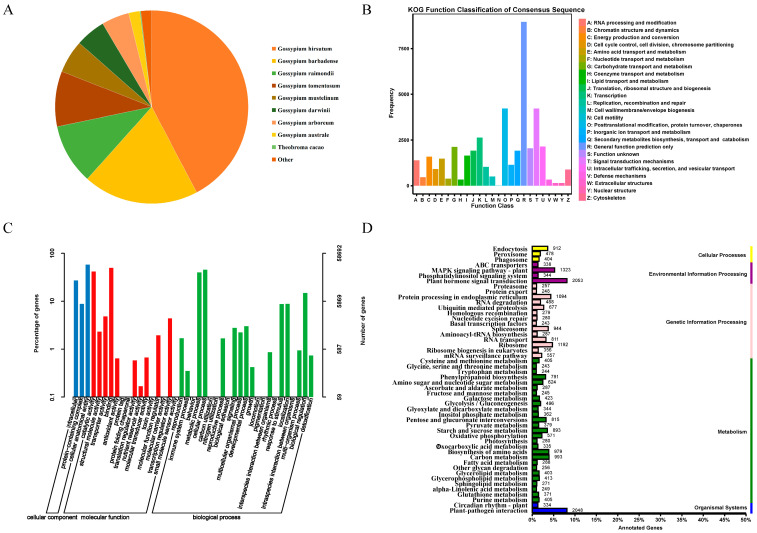
Unigene annotations. (**A**): Distribution of homologous species based on Nr annotations. (**B**): Unigene KOG database comparison results. (**C**): Unigene GO annotation entries. (**D**): Unigene KEGG annotation category map.

The total RNA quality of the samples was examined, and it was determined that the concentration and integrity of the 42 samples met the requirements for RNA-Seq sequencing cDNA library construction (Table S1). A total of 288.63 Gb of clean data were obtained from the sequencing library of the 42 samples. The clean data for each sample amounted to 5.76 Gb, with a GC content above 43.16% and a Q30 base percentage above 93.77%. The sequencing results met the experiment’s requirements and could be further analyzed (Table S2). The clean data for each sample were aligned to the reference genome, TM-1, and 90.49–97% of the clean reads for the 42 samples were aligned to this reference genome, of which the number of reads that aligned to a unique position accounted for 85.96–93.62% of the clean reads, and the number of reads aligned to multiple positions accounted for 3.15–4.53%. In total, 4.53% (Table S3) of the RNA-seq data were assembled to obtain 77,085 unigene sequences. The NCBI Nr annotation results revealed that 42.38% of the unigenes were most similar to *G. hirsutum*, while 19.34% of the unigenes were similar to *Gossypium barbadens*, reflecting the kinship between the two cotton genera (Figure 1A).

To shed more light on the functional allocation of cotton genes, an in-depth comparison and classification of the assembled unigenes was conducted against the KOG database. This analysis revealed that 11.62% of these unigenes were grouped under general function prediction, and the next highest grouping was under post-translational protein modification. In total, 3.41% of the unigenes were linked to transcriptional processes, and 2.78% were involved in intracellular transport, secretion, and vesicular transport. Carbohydrate transport and metabolism accounted for 2.76%, and translation, ribosome structure, and biogenesis accounted for 2.50%. Secondary metabolite biosynthesis, transport, and catabolism constituted 2.49%, and 2.14% of the unigenes were tied to lipid transport and metabolism. In total, 2.07% of the unigenes were related to energy production and conversion; amino acid transport and metabolism; RNA processing and modification; inorganic ion transport and metabolism; replication, recombination, and repair; cell cycle control; cell division; and chromosome division. Cytoskeleton-related unigenes constituted less than 2%. Cell wall/membrane/envelope biogenesis, chromatin structure and dynamics, nucleotide transport and metabolism, auxin transport and metabolism, defense mechanisms, nuclear structure, extracellular structure, and cell-motility-related unigenes accounted for less than 1%. In addition, another 2.66% remained functionally unclassified (Figure 1B).

The GO annotation results showed that 58,692 Unigenes were assigned annotation entries, and cellular components were annotated to the most cellular anatomical entity entries (34,059 entries), accounting for 61.47%. The molecular functions of binding and catalytic activity were the most annotated, with 29,382 and 24,615 entries, respectively, accounting for 46.44% and 38.90% of all annotations. This was followed by transporter activity, with 2830 annotations (4.47%). The biological processes that had the most annotations were the cellular process and the metabolic process, with 26,883 and 23,627 annotations, respectively; in addition, biological regulation, response to stimulus, and localization had more than 5000 annotations (Figure 1C).

The KEGG annotation results showed that all the unigenes were annotated as cellular processes (1752), environmental information processing (3406), genetic information processing (7403), metabolism (12,167), and organic systems (2382). The second tier of signal transduction and environmental adaptation had the most annotations for phytohormone signaling and plant–pathogen interactions, with 2053 and 2048 annotations, respectively, followed by the MAPK signaling pathway in signal transduction (1323), ribosomes (1192), endoplasmic reticulum protein information processing in genetic information processing (1094), amino acid biosynthesis (979), carbon metabolism (993), and transcriptional shearing in genetic information processing (944). The remaining annotation entries had fewer than 900 unigenes (Figure 1D).

### 3.2. Differentially Expressed Gene Analysis

**Figure 2:**
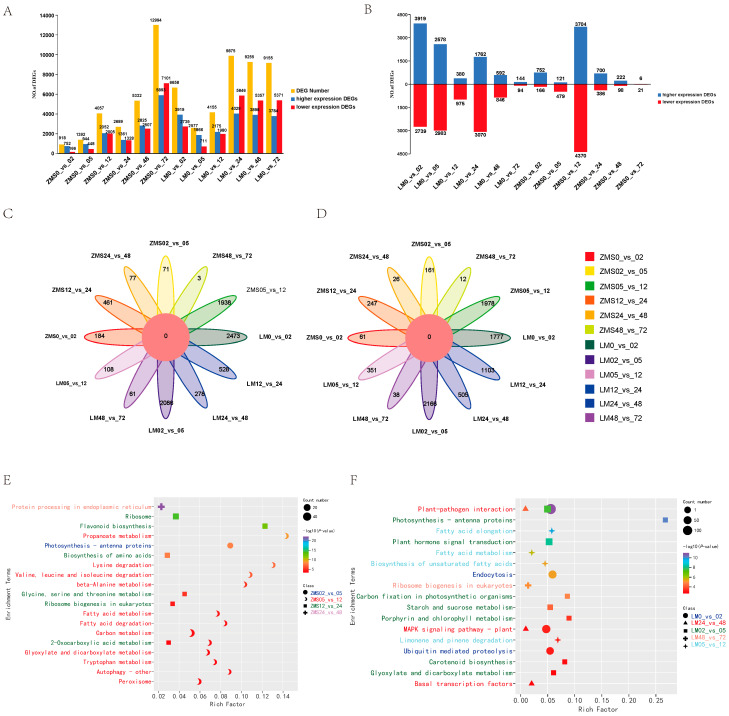
Analysis of DEGs. (**A**): Number of DEGs between two cotton cultivars in the control and stress treatments. (**B**): Number of DEGs between adjacent sampling time points after the stress treatment. (**C**): Common and specific highly expressed DEGs in the 12 comparison groups. (**D**): Common and specific poorly expressed DEGs in the 12 comparison groups. (**E**): KEGG enrichment analysis of highly expressed DEGs in the ZMS comparison groups. (**F**): KEGG enrichment analysis of highly expressed DEGs in the LM comparison group.

In order to clarify the responses of different salt-tolerant land cotton varieties to salt stress, DEGs were identified using FDR < 0.05 and Fold Changes ≥ 2. Both ZMS and LM produced a large number of DEGs after being subjected to salt stress, and the number of DEGs in ZMS continued to increase within 72 h, but gene mobilization was not strong in the pre-stress period. On the contrary, LM showed more active and rapid expression in the pre-salt-response period, which gradually leveled off after 24 h (Figure 2A). During the time intervals of 2 h, 5 h, 12 h, 24 h, 48 h, and 72 h, 3919, 2578, 380, 1762, 592, and 144 DEGs were found to be highly expressed in LM, and 752, 121, 3704, 700, 222, and 6 DEGs were found to be highly expressed in ZMS, suggesting that LM at 2 h, 5 h, and 24 h and ZMS at 12 h had specific response patterns (Figure 2B). The analysis of the DEGs among different comparison groups revealed that although there were commonalities at adjacent time points, most of the DEGs corresponded to the specific differential expression at that time point; moreover, the common parts were not screened in all the higher expression and lower expression DEG control groups (Figure 2C,D). Further GO enrichment analysis of these unique DEGs showed that overall (Figure S1), genes were mobilized more in biological processes and molecular functions than in cellular components after stress. Time intervals of 2 h, 5 h, 12 h, 24 h, 48 h, and 72 h LM were significantly enriched in DNA-binding transcription factor activity (GO:0003700), plasma membrane (GO:0005886), endoplasmic reticulum chaperone complex (GO:0034663), photosystem II (GO:0009523), negative regulation of DNA recombination Gene Ontology (GO:0045910), and water channel activity (GO:0015250). ZMS was most significantly enriched in the blue light signaling pathway (GO:0009785), photosystem II (GO:0009523), ribosome (GO:0005840), structural constituent of ribosome (GO:0003735), protein complex oligomerization (GO:0051259), and kinase activity (GO:0016301). This result demonstrates that LM responds more rapidly to salt stress. In the early stage, the defense mechanism was initiated by the activation of transcription factors to elicit downstream responses, while the plasma membrane structure was regulated, maintaining intracellular ion levels via ion channel control. In the later stage, LM accumulated organic matter through photosynthesis, and the activity of water channels was enhanced to minimize the cellular damage caused by osmotic stress. ZMS responded to salt stress in a simpler way by enhancing photosynthesis to resist environmental stress in the early stage and then focusing on protein synthesis in the later stage. This might be related to the maintenance of osmotic homeostasis. The specific regulatory mechanisms of LM and ZMS in response to salt stress led to a difference in their salt tolerance.

In order to reveal the temporal pattern changes in response to salt stress in different genotypes of cotton, KEGG enrichment analysis was performed on specific DEGs in each control group, and the 20 most significant pathways were retained (Table S4). ZMS was significantly enriched in one pathway, twelve pathways, six pathways, and one pathway at 5 h, 12 h, 24 h, and 48 h, indicating that the response pattern was more complex at 12 h. The most significant enrichment was for protein processing in the endoplasmic reticulum at 48 h containing 25 DEGs; in addition, 12 h and 24 h involved a large number of DEGs related to amino acid anabolism and fatty acid metabolism, which may be related to cellular membrane damage and repair after salt stress (Figure 2E). The LM was significantly enriched in the metabolic pathways at 2 h, 5 h, 12 h, 48 h, and 72 h, indicating that although the number of LM DEGs leveled off after 24 h, it was regulated and maintained by different metabolic networks over time for the plants’ adaptation to the salt environment and engagement in normal life activities therein (Figure 2F).

**Figure 3:**
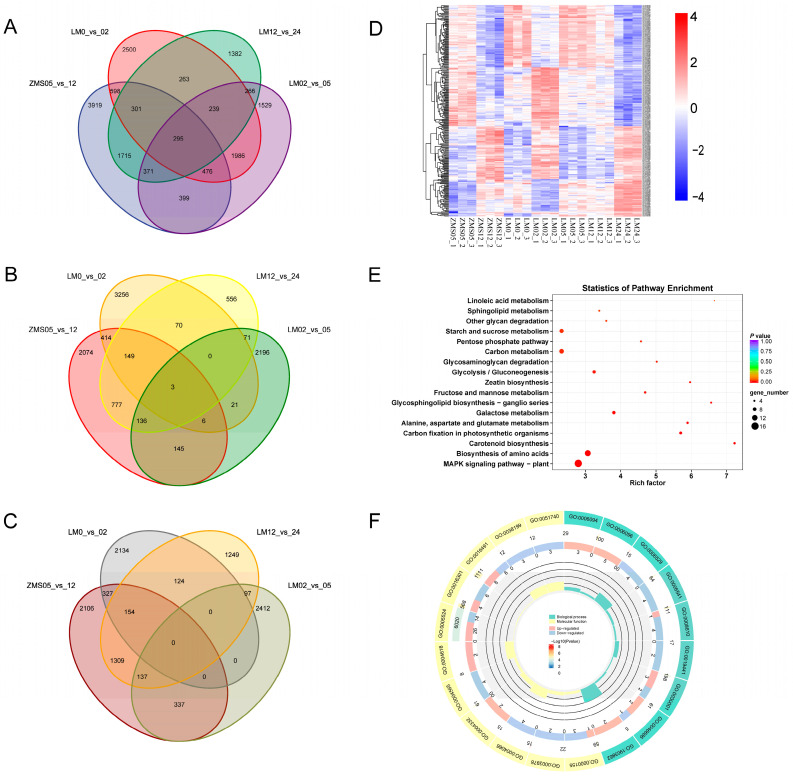
Salt tolerance-related DEG analysis. (**A**): Comparison of the number of DEGs in the four comparison groups. (**B**): Comparison of the number of highly expressed DEGs in the four comparison groups. (**C**): Comparison of the number of poorly expressed DEGs in the four comparison groups. (**D**): Clustering heatmap of co-expressed DEGs based on FPKM. (**E**): KEGG enrichment analysis of co-expressed DEGs. (**F**): GO enrichment analysis of co-expressed DEGs.

There were numerous gene expression variations in the four comparison groups, i.e., ZMS05_vs._12, LM0_vs._02, LM02_vs._05, and LM12_vs._LM24, and selecting the overlapping part allowed us to reduce the number of redundant genes, which was conducive to the in-depth investigation of salt-tolerant genes. There were 295 genes common to the four groups, and these might be closely related to the salt tolerance response of cotton (Figure 3A). There were 239 genes in the LM comparison group that were shared at three time points but were not found in ZMS. These genes might be responsible for the formation of salt tolerance characteristics in LM. Upon comparing the highly and poorly expressed genes separately, it was found that only three genes were commonly highly expressed in the four groups (Figure 3B), and no common poorly expressed genes were found (Figure 3C). This suggests that LM and ZMS exhibit different response patterns in regard to salt stress.

Heat mapping was performed for 295 genes to clarify their trends (Figure 3D). Although these genes were common to both LM and ZMS, there was still a portion of genes with different response patterns in the two cultivars. Overall, the DEGs showed the same expression trend in LM05 and LM12, suggesting that the metabolic regulatory network associated with these genes may provide continuous feedback regarding salt stress during this period. Based on the results of GO and KEGG enrichment, these genes may be involved in various metabolic pathways such as MAPK signaling, amino acid biosynthesis, carotenoid biosynthesis, and carbon fixation in photosynthetic organisms through amino acid catabolism, oxidative phosphorylation, and phytohormone sensing, and may play a role in the resistance to salt stress (Figure 3E,F; Table S5).

### 3.3. WGCNA Analysis

In order to screen the genes associated with playing a key role in salt tolerance, strict screening conditions were set for the DEGs generated from samples within four time periods: Fold Change ≥ 5 and FDR < 0.01 for ZMS05_vs._12, LM0_vs._02, and LM02_vs._05, LM12_vs._LM24. Finally, 2742 genes were used as the background to construct a weighted gene co-expression network. The weight values were calculated using the WGCNA package. Here, when the soft threshold β = 17, the gene expression matrix conformed to the scale-free network (Figure S2).

**Figure 4:**
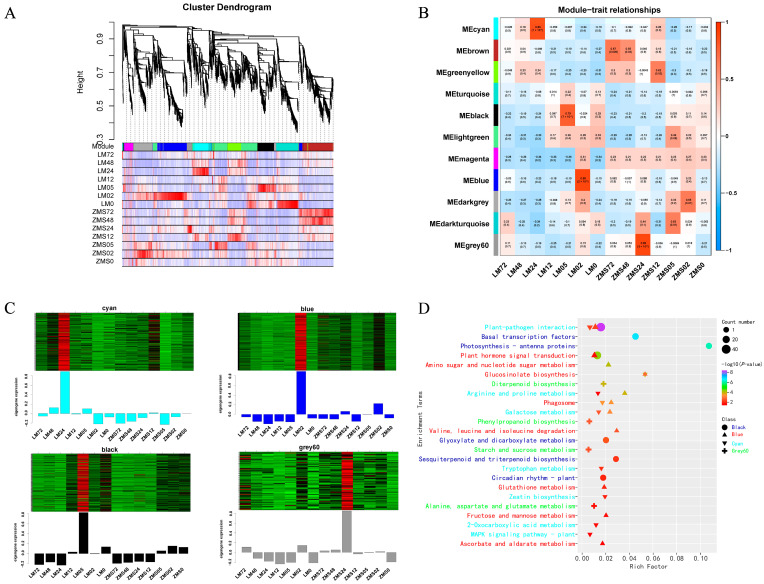
Weighted gene co-expression network analysis. (**A**): Clustering and module delineation. (**B**): Module-and-trait correlation. (**C**): Heatmap of gene expression within the modules. (**D**): Analysis of KEGG enrichment of genes in modules.

All genes were divided into eleven modules (Figure 4A), and the number of genes within the modules ranged from 47 to 448, with four modules significantly correlating with the traits noted here: the Cyan module correlated with LM24 (R^2^ = 0.86, *p* = 1 × 10^−4^), with 218 genes; the Black module correlated with LM05 (R^2^ = 0.79, *p* = 7 × 10^−4^), with 243 genes; the Blue module correlated with LM02 (R^2^ = 0.89, *p* = 2 × 10^−5^), with 443 genes; and the Grey60 module correlated with ZMS24 (R^2^ = 0.89, *p* = 3 × 10^−5^), with 67 genes (Figure 4B). The heatmap developed based on the log FPKM values reflected the unique expression patterns of the four specific modules corresponding to the time points (Figure 4C).

KEGG enrichment analysis was performed on the genes in the modules. Only the pathways with a *p* value < 0.05 were retained for plotting (Figure 4D, Table S6). Plant–pathogen interaction pathways were co-enriched in the Black, Blue, and Cyan modules, suggesting their importance in the early and late stages of the salt stress response in LM. Plant hormone signal transduction, glucosinolate biosynthesis, arginine and proline metabolism, the phagosome, and galactose metabolism were co-enriched in two modules, confirming their critical roles in the salt stress response. In addition, the enrichment results for the ZMS-associated Grey60 module were completely different from those for the other three modules, showing only four pathways: diterpenoid biosynthesis; phenylpropanoid biosynthesis; starch and sucrose metabolism; and alanine, aspartate, and glutamate metabolism represented the response strategies of ZMS to salt stress. The expression pattern analysis of these modules can help to further reveal the regulatory mechanism of salt tolerance in cotton.

The transcription factor prediction of salt tolerance-related genes in the four modules resulted in a total of one hundred and fourteen *TFs* (Table S7), including twenty WRKYs, seventeen ERFs, sixteen NACs, thirteen bHLHs, seven bZIPs, and six MYBs (Figure S3). They play important regulatory roles in the salt stress response, as evidenced by their higher expression in the corresponding periods.

### 3.4. Module Visualization and Screening of Hub Genes

**Figure 5:**
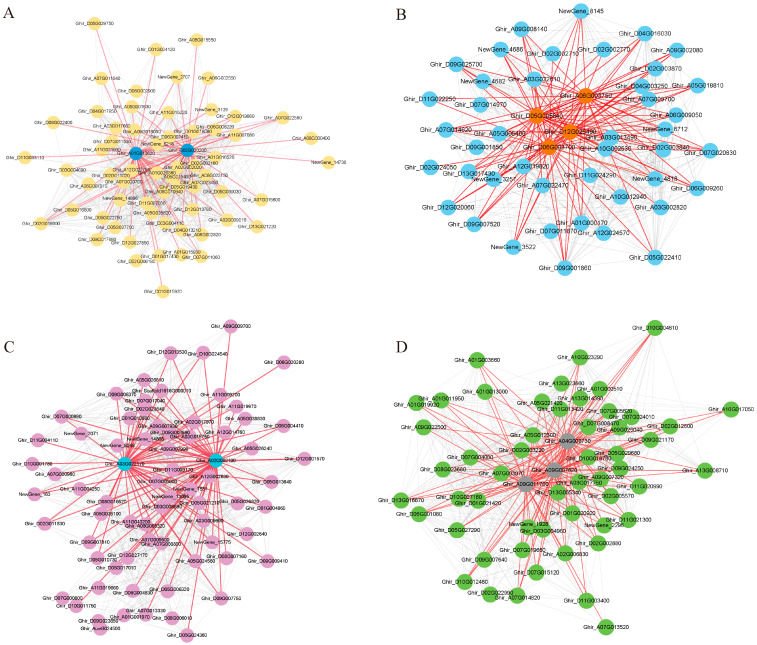
Co-expression network map of genes within the salt tolerance-related module. (**A**): Black module, blue circles represent hubgene. (**B**): Blue module, orange circles represent hubgene. (**C**): Cyan module, blue circles represent hubgene. (**D**): Grey60 module, grey circles represent hubgene. Each node represents a gene and the lines denote the connections between genes.

The mining of key genes in the co-expression modules allowed us to find important regulatory genes. Based on the weight values and connectivity of the genes in the modules, members of the four modules with KME values greater than 150 were visualized using Cytoscape. The hub gene screening parameter values were set as follows: weight > 0.35 and degree > 5. Eleven central genes were screened from the four salt tolerance-related modules (Figure 5, Table 1). *Ghir_A01G013620* (COR2) and *Ghir_D02G020200* (EXL5) were considered the major regulatory genes in the Black module. *Ghir_D05G005640*, *Ghir_A06G003750*, and *Ghir_D06G003700* are related to MDIS1-interacting receptor-like kinase 2 (MIK2); these three genes, along with *Ghir_D12G026490* (WRKY30), play key roles in the Blue module. *Ghir_A03G022170* (ATJ11) and *Ghir_A02G002100* (SBP2) occupy a pivotal position in the gene co-expression network of the Cyan module. *Ghir_A04G008730* (ASL), *Ghir_A09G007820* (SKIP27), and *Ghir_A09G011700* (F6′H1) are the key genes in Grey60 module. Salt stress affected the synergistic expression of these genes, and their role in cotton salt tolerance needs to be further verified.

### 3.5. DEG Validation Using qRT-PCR

**Figure 6:**
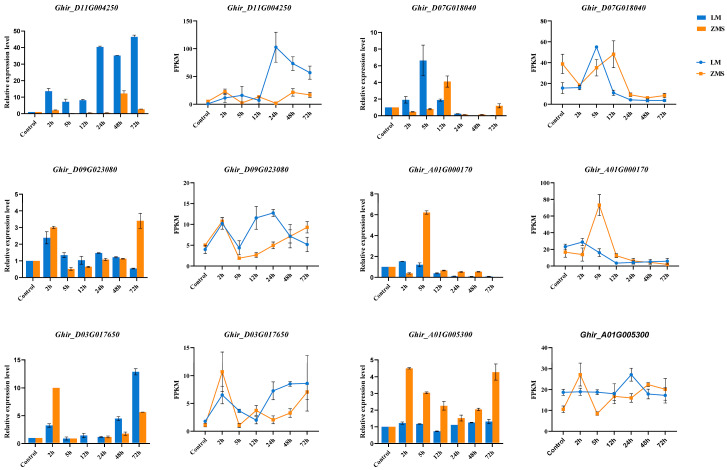
qRT-PCR validation of DEGs. The histograms show the relative expression of the DEGs, the line graphs show the DEG transcriptome expression profiles.

The reliability of the transcript expression profiles obtained via RNA-seq was verified using qRT-PCR. Six DEGs were randomly selected to design specific primers for amplification in 42 samples (Figure 6, Table S8). The QRT-PCR results showed that the expression levels of the selected genes changed significantly under salt stress, and *Ghir_D11G004250* was consistently highly expressed in LM at multiple time points. According to the gene annotation results, *Ghir_D11G004250* belongs to the bHLH transcription factor family, demonstrating the potential critical role of this gene in salt tolerance. *Ghir_A01G000170* and *Ghir_D07G018040* were annotated as NAC domain-containing protein 35 and Chlorophyll a-b binding protein 151, respectively, and these two genes were significantly highly expressed within 12 h of stress induction, suggesting a role in the prophylactic response. In addition, the expression patterns of *Ghir_D09G023080*, *Ghir_D03G017650*, and *Ghir_A01G005300* revealed the association of ETR2, At4g16563, and HBDA with salt tolerance according to the gene annotation results. Overall, the qRT-PCR results were positively correlated with the gene change trends revealed via RNA-seq, which verified the reliability of the RNA-seq data.

## 4. Discussion

### 4.1. RNA-Seq Mining of Salt Tolerance Genes

Plants’ responses to adverse environmental conditions involve complex molecular mechanisms involving the synergistic action of many genes. These mechanisms precisely and specifically regulate cellular biological response processes in different tissues and response phases to maintain the homeostasis and ion balance of the internal environment to allow the acquirement of adaptive resistance. A comparative time point experimental design enables the systematic measurement of changes in the expression levels of most genes in space and time, a process that can decipher the dynamic and coordinated roles of genes in complex regulatory networks when plants respond to unfavorable conditions [28,29]. István Szádeczky-Kardoss performed comparative time point transcriptome sequencing on wild-type and TFIIS-1 mutant *Arabidopsis thaliana* specimens, demonstrating that TFIIS is dynamically regulated during heat stress and sustains transcription to support proteotoxic stress and cellular pathway (including photosynthesis) processes [30]. This finding has also been reported for cotton resistance to salt stress. Previous transcriptome analyses of non-transgenic and transgenic *ScALDH21* cotton at 0 d, 2 d, and 5 d after salt stress showed that the *ScALDH21* gene enhances cotton resistance to salt stress by stimulating the expression of genes related to stress responses, enhancing photosynthesis and improving carbohydrate metabolism [31]. In this study, ZMS and LM were used as salt-sensitive and salt-tolerant varieties, respectively. By sequencing the comparative time point transcriptomes of the salt-tolerant differential cotton cultivars, we aimed to find the common response pathways and specific regulatory pathways involved in salt stress and clarify the effects of each metabolic regulatory network on the salt tolerance characteristics. The transcriptome sequencing results showed that 288.63 Gb of clean data were obtained from 42 sequenced libraries. The amount of clean data for all the samples reached 5.76 Gb, with a GC content above 43.16% and a Q30 base percentage above 93.77%. At the same time, qRT-PCR was used to detect the gene expression profiles of the transcriptome, and the above results provide a strong basis for further analysis and mining using RNA-seq data.

### 4.2. Metabolic Networks Regulate Cotton’s Resistance to Salt Stress

Both LM and ZMS mobilized numerous differentially expressed genes to help them acclimatize to the high-salt environment within 72 h after salt stress treatment. The KEGG enrichment results indicated the variability of metabolic regulation patterns at different time stages. The up-regulated, differentially regulated genes at the initial stage of the LM stress response were mainly enriched in photosynthesis-related antenna proteins. It has been demonstrated that plants undergoing photosynthesis had elevated expression during salt stress [32], and ensuring the stability of the photosystem helps to enhance the tolerance of plants [33]. At 12 h, lysine degradation; valine, leucine, and isoleucine degradation; beta-alanine metabolism; glyoxylate and dicarboxylate metabolism; tryptophan metabolism; and autophagy, as well as the peroxisome, fatty acid metabolism, fatty acid degradation, and carbon metabolism, were significantly enriched, reflecting that at this point, the major modes of regulation in plants involve amino acid biosynthesis, membrane lipid remodeling, autophagy, and ROS scavenging. Multiple types of amino acid synthesis and accumulation are detected in most abiotic stresses [34,35,36]. It is widely recognized that the accumulation of some specific amino acids is associated with the enhancement of plant resistance [35,37], However, there is no consensus on the potential mechanism of action of these amino acids, which may act as precursors for the synthesis of other secondary metabolites or regulate osmotic homeostasis due to changes in amino acid content induced by protein turnover under adverse conditions [38]. According to existing studies, fatty acid degradation leads to a decrease in membrane fluidity [39,40], further limiting the uptake of Na+ and Cl− by relevant transport proteins to maintain osmotic homeostasis [41]. Additionally, reduced membrane fluidity reduces membrane sensitivity, helping to maintain integrity [42]. In this study, the fatty acid metabolic pathway changes were consistent with those observed in *Mesembryanthemum crystallinum* L., *Spartina patens*, and soybean under haline conditions [43,44,45], revealing an important regulatory mechanism of membrane homeostasis in salt stress. Many studies have shown that increased unsaturated fatty acid content contributes to stress tolerance [46]. It has been reported that this mechanism of action occurs due to the fact that unsaturated fatty acids alleviate salt-stress-induced PSII photoinhibition, safeguarding photosynthetic processes [47]. Autophagy is a conserved evolutionary cellular degradation process, playing a key role in plants’ stress responses [48]. Similar to the results reported for cucumber and poplar, the results for *Arabidopsis* suggested that salt stress induces the expression of autophagy genes to produce autophagosomes, which alleviate stress by accelerating the turnover of oxidized proteins in cells [48,49,50]. Peroxisomes are involved in ROS metabolism and regulate cellular redox levels, playing an important role in defense against oxidative stress [51]. The significant enrichment of peroxisome metabolism observed in the presented study was also found in regard to wheat, rice, and allotriploid *Populus cathayana* [52,53,54]. In addition to the similarity in regulation in ZMS, the regulatory mechanisms of LM with respect to salt stress include plant–pathogen interactions, plant hormone signal transduction, the MAPK signaling pathway, and carotenoid biosynthesis. Plant–pathogen interactions are activated through PAMP-triggered immunity (PTI) and effector-triggered immunity (ETI) [55]. The activation of PTI and ETII involves MAPK signaling [56], which is regulated by the major phytohormone abscisic acid (ABA) [57] and activates downstream responses through numerous *TFs* [58]. In salinity-treated oat, researchers also detected plant–pathogen interactions, plant hormone signal transduction, and MAPK signaling pathway activity [59], consistent with the results of the present study, revealing the integrated role of these signaling pathways in salt stress. In addition, the co-DEGs of LM and ZMS were mainly enriched in carbohydrate metabolism, a process that involves carbon allocation under stress [60]. C-labeled carbon partitioning showed that the aboveground parts of saline plants receive more carbon partitioning than belowground parts under salt stress to improve the defense system [61]. In perennial ryegrass, both salt-sensitive cultivars and salt-tolerant cultivars’ salt stress responses involved carbohydrate metabolism and carbon partitioning [62], consistent with the results of this study. The involvement of ABA in stress acclimation has been reported in most plants [63,64,65], and the biological functions of ABA are mainly manifested in the control of stomatal closure [66], the regulation of osmotic homeostasis [67], and crosstalk stress signaling [68], all of which play important roles. The accumulation of carotenoids as ABA synthesis precursors is also crucial in salt stress [69]. The overexpression of the carotenoid biosynthesis genes *LcPDS*, *LcZDS*, and *LcCRTISO* enhances salt tolerance in tobacco [63].

### 4.3. TFs Reveal Salt Tolerance Strategies in Cotton

We identified several *TFs* involved in the salt tolerance response, namely, WRKY, ERF, NAC, bHLH, bZIP, and MYB, which may play defense roles by regulating the expression of downstream genes. WRKY, as one of the largest families of *TFs* in plants, is involved in abiotic stress response processes through phytohormone signaling and ROS scavenging pathways [70,71]. WRKY TFs are involved in hormonal processes, mainly in the ABA signaling pathway. Additionally, the overexpression of *AtWRKY25* and *AtWRKY33* increased salt tolerance in *Arabidopsis*, while 31 and 208 potential downstream targets related to ABA were identified [72]. Other studies have shown that salt tolerance is enhanced under stress conditions by accumulating more endogenous ABA in transgenic lines [73,74,75,76,77]. The overexpression of *IbWRKY2* and *HbWRKY83* in *Arabidopsis* enhances salt tolerance through improving ROS scavenging [78,79]. ERF, an AP2/ERF superfamily protein, plays an important role in plant defense responses by targeting and regulating Na+/K+ transporter genes and antioxidant enzyme genes to maintain ionic and ROS homeostasis. The *MdERF106* gene of *Malus domestica* promotes the downstream expression of *MdSOS1*, which, in turn, promotes Na+ efflux [80]. LcERF056 enhances salt tolerance in *Lotus corniculatus* by directly up-regulating the ROS-related genes *LcLTP*, *LcPrx*, and *LcRP* [81]. All the above evidence supports the notion of a regulatory strategy of ERFs in salt stress. The positive role of NAC under salt stress has been characterized in multiple crops [82,83,84]. The overexpression of *CarNAC4* enhances the expression of the downstream stress-responsive genes *RD29A*, *ERD10*, *COR15A*, *COR47*, *KIN1*, and *DREB2A* to improve salt tolerance [84]. The up-regulated expression of bHLH under salt stress confers plant tolerance and plays a significant role in Na+/K+ ion transport, ROS scavenging, and osmoregulation [85,86,87]. A large body of evidence suggests that bZIP is involved in an ABA-dependent stress signaling pathway, activating downstream resistance-related genes by binding to the ABA response element ABRE, and that downstream genes regulate salt tolerance in terms of secondary metabolites, osmoregulation, and ROS scavenging [88,89,90]. The defense of MYB against salt stress in plants is mainly reflected in the induction of synthesis secondary metabolites, including anthocyanins, flavonoids, and lignans [91,92]. The accumulation of anthocyanins and flavonoids under stress promotes endogenous ROS scavenging and reduces oxidative damage [91,93,94,95]. Lignin is an important component of the cell wall, and lignin deposition contributes to the strengthening of the physical function of the cell wall and plays an important role in salt acclimatization [91,96]. The regulatory role of MYB in salt tolerance has been demonstrated in *Arabidopsis*, maize, and rice [97,98,99]. All six *TFs* mentioned above were identified in our study, providing a reference basis for analyzing the salt tolerance strategy of cotton.

### 4.4. Salt Stress Affects Hubgene Expression

We screened 11 genes for changes in expression under salt stress by analyzing the gene interaction network within salt tolerance-related modules. According to the results of previous studies, *COR2* is a downstream-regulated gene of the WRKY transcription factor family, an enzyme that plays a key role in the biosynthesis of benzylisoquinoline alkaloids (BIAs) [100,101]. There is still a large gap in research on the involvement of BIAs in abiotic stresses, and we have compiled the possible roles of BIA from only a few reports. Li isolated ten alkaloid species, two of which, both BIAs, exhibited antioxidant activity that was closely related to ROS and free-radical-mediated oxidative stress [102]. Similar reports mention that BIA accumulation can effectively alleviate oxidative stress and improve plant salt resistance [103]. Based on this evidence, we hypothesized that *COR2* plays a role in synthesizing BIAs to balance ROS levels. In addition, a little-known fact about the function of EXL5 is that it is significantly up-regulated after metal contamination in *Arabidopsis*, so it could serve as a biomarker for metal stress [104]. Differential expression of EXL5 was similarly detected under Fe stress [105]. Although there are no reports on EXL5 in relation to salt stress, based on the similarity of plant defense mechanisms under abiotic stresses [106], these findings may provide a reference for further studies on the mechanism of action of EXL5 in response to salt stress. MIK2 is a membrane receptor that senses extraplasmic environmental stresses and triggers immunity [107]. MIK2 mutants are defective in regard to both salt stress tolerance and cell wall integrity [108]. Another study showed that in NaCl-stress-induced *Arabidopsis*, MIK2-1 accumulated more dry weight than the wild type, and MIK2 expression enhanced biomass assimilation [109]. All of this evidence demonstrates that MIK2 is required for salt tolerance. *WRKY30*, belonging to the WRKY transcription factor superfamily, activates defense responses by binding to multiple stress genes. The overexpression of *AtWRKY30* enhances salt tolerance in *Arabidopsis* [110]. The positive role of WRKY30 in salt stress was also characterized in *Michx* and grape [111,112]. ATJ11 is a molecular chaperone heat-shock protein (DnaJ/Hsp40). A study on GsJ11, the homologue of ATJ11 in soybean, showed that GsJ11 was highly induced in the presence of NaHCO3 within 3 h. The mutant line was less tolerant to NaHCO3 than the wild strain, and the stress-responsive genes in the overexpression strain had higher levels of transcription [113]. Another study characterized the regulatory capacity of ATJ11 in chloroplast redox through a global stress response triggered by an ATJ11 knockout mutant [114]. These reports provide a reference point for studying the role of ATJ11 in salt tolerance. SBP2, a selenium-binding protein, is involved in the process of selenium (Se) synthesis and accumulation. Under salt stress, Se accumulation can regulate the ROS pathway and osmotic metabolism and thus protect wheat from oxidative damage [115]. The same conclusion was reached in regard to soybean, to which Se conferred salt tolerance [116]. Argininosuccinate lyase (ASL) catalyzes the breakdown of argininosuccinate to arginine and plays an important role in arginine metabolism. The catalytic role of ASL allows plants to amass sufficient nitrogen reserves under stress conditions, and these reserves are further allocated to defense regulatory mechanisms and help to refine a plant’s energy conversion in unfavorable environments [117]. The F-box protein SKIP27 identified in this study was also shown to be induced by salt stress in another report [118], suggesting that this gene may play an important role in the salt tolerance response, and its function needs to be further verified. F6′H1 is an essential enzyme for coumarin biosynthesis [119]. Coumarin synthesis effectively improves iron uptake by plants in saline soils, helping to maintain normal life activities under salt stress [119]. The expression of *GmF6′H1* in soybean is significantly salt-inducible, and the overexpression of *GmF6′H1* in *Arabidopsis* enhanced salt tolerance [120]. In summary, the 11 genes screened via the gene interaction network in this study may function in the salt stress response, and their specific mechanism of action needs to be verified in future experiments.

## 5. Conclusions

In this study, RNA-seq analysis of cotton revealed certain commonalities and specificities in the salt stress response of cotton cultivars with different sensitivities. Photosynthesis, amino acid biosynthesis, membrane lipid remodeling, autophagy, and ROS scavenging are co-regulated pathways of both. Salt-tolerant cultivars have better performance in regard to metabolic pathways such as plant–pathogen interactions, plant hormone signal transduction, the MAPK signaling pathway, and carotenoid biosynthesis. Four salt tolerance-related modules were screened using WGCNA; they contained 114 *TFs*, of which WRKY, ERF, NAC, bHLH, bZIP, and MYB were important regulators in response to salt stress. Gene interaction network analysis identified 11 key genes: *Ghir_A01G013620*, *Ghir_D02G020200*, *Ghir_D05G005640*, *Ghir_A06G003750*, *Ghir_D06G003700*, *Ghir_D12G026490*, *Ghir_A03G022170*, *Ghir_A02G002100*, *Ghir_A04G008730*, and *Ghir_A09G007820*. They are hypothesized to function in the salt stress response.

## Figures and Tables

**Table 1:** Functional annotation of hub genes in the modules.

Modules	Gene ID	Gene Name	Description
Black	*Ghir_A01G013620*	COR2	Non-functional NADPH-dependent codeinone reductase 2
	*Ghir_D02G020200*	EXL5	Protein EXORDIUM-like 5
Blue	*Ghir_D05G005640*	MIK2	MDIS1-interacting receptor-like kinase 2
	*Ghir_A06G003750*	MIK2	MDIS1-interacting receptor-like kinase 2
	*Ghir_D06G003700*	MIK2	MDIS1-interacting receptor-like kinase 2
	*Ghir_D12G026490*	WRKY30	Probable WRKY transcription factor 30
Cyan	*Ghir_A03G022170*	ATJ11	Chaperone protein dnaJ 11, chloroplastic
	*Ghir_A02G002100*	SBP2	Selenium-binding protein 2
Grey60	*Ghir_A04G008730*	ASL	Argininosuccinate lyase
	*Ghir_A09G007820*	SKIP27	F-box protein SKIP27
	*Ghir_A09G011700*	F6′H1	Feruloyl CoA ortho-hydroxylase 1

## Data Availability

The relevant data are contained within this paper and its supporting files. The sequencing data generated in this study have been uploaded to the National Center for Biotechnology Information Database under BioProject PRJNA1134120.

## Supplementary Materials

The following supporting information can be downloaded at: https://www.mdpi.com/article/10.3390/genes15091176/s1, Table S1: Assessment of the integrity and quality of RNA samples; Table S2: Statistical results regarding the sequencing data; Table S3: Comparison of sequencing data with reference sequences; Table S4: Analysis of specific DEGs’ KEGG enrichment in ZMS and LM; Table S5: KEGG enrichment in the co-DEGs of LM and ZMS; Table S6: Results for KEGG enrichment of modules significantly correlated with traits; Table S7: Transcription factors associated with salt tolerance; Table S8: Primers used in qRT-PCR; Figure S1: ZMS and LM unique DEGs GO enrichment analysis; Figure S2: Softpower plot; Figure S3: Transcription factors in the salt tolerance module.
