# Supplementary figures and images for: RNA-Seq and WGCNA Analyses Reveal Key Regulatory Modules and Genes for Salt Tolerance in Cotton
Source: Genes (Basel). 2024 Sep 7;15(9):1176. doi: 10.3390/genes15091176 (PMC11431110; doi:10.3390/genes15091176)

LM0\_vs\_02

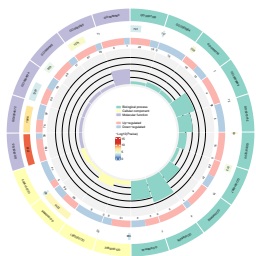

LM02\_vs\_05

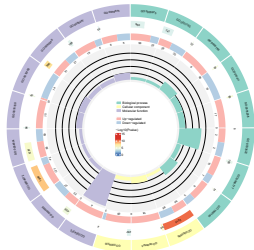

LM05\_vs\_12

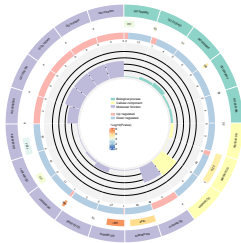

LM12\_vs\_24

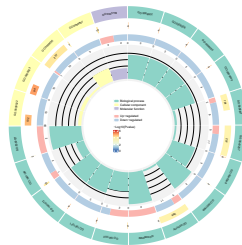

LM24\_vs\_48

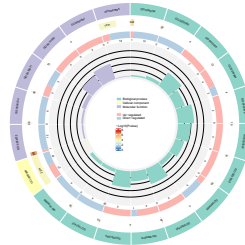

LM48\_vs\_72

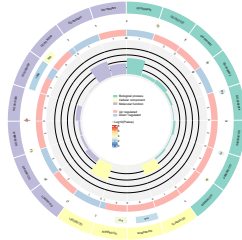

ZMS0\_vs\_02

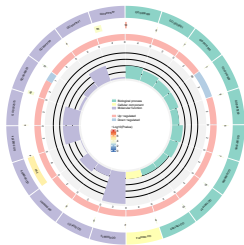

ZMS02\_vs\_05

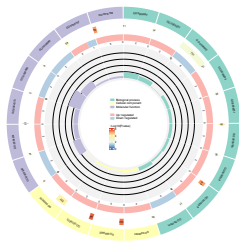

ZMS05\_vs\_12

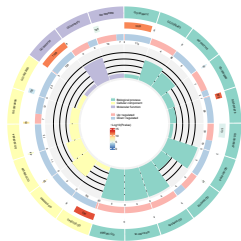

ZMS12\_vs\_24

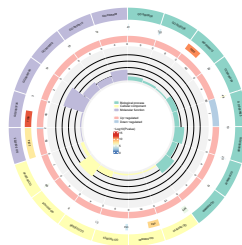

ZMS24\_vs\_48

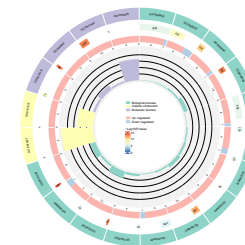

ZMS48\_vs\_72

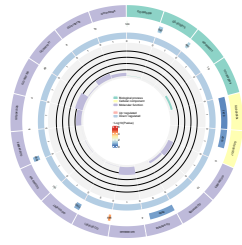

Supplement: Supplementary file 1 [file genes-15-01176-s001.zip › Figure S1. ZMS and LM unique DEGs GO encirhment analysis.pdf]

### Scale independence

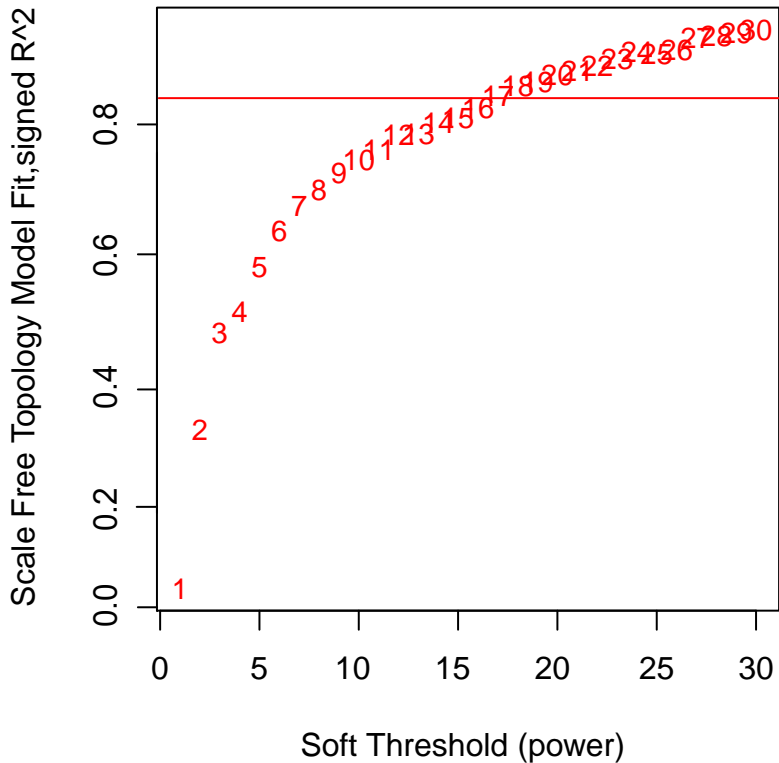

### Mean connectivity

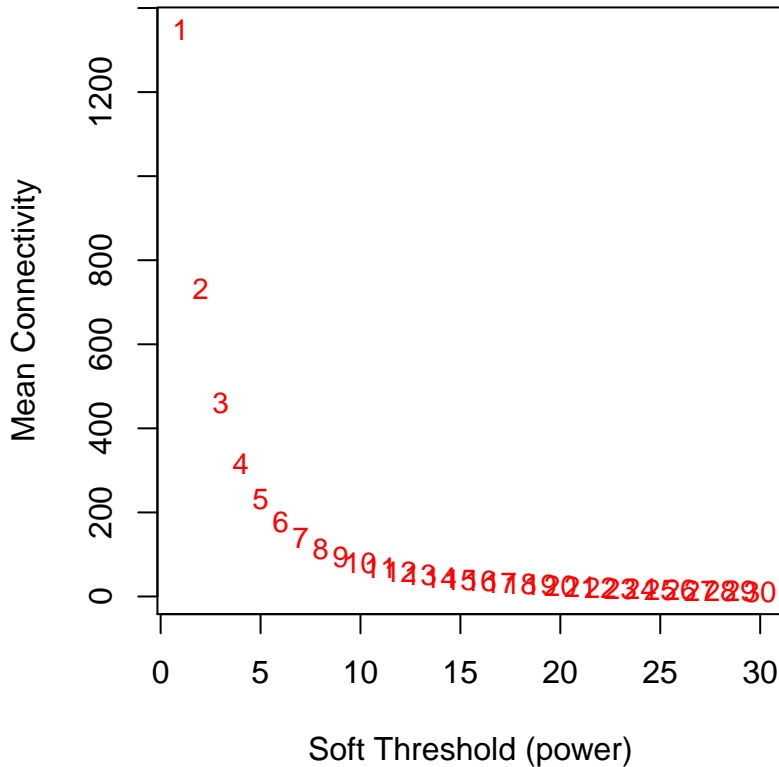

Supplement: Supplementary file 1 [file genes-15-01176-s001.zip › Figure S2. Softpower plot.pdf]

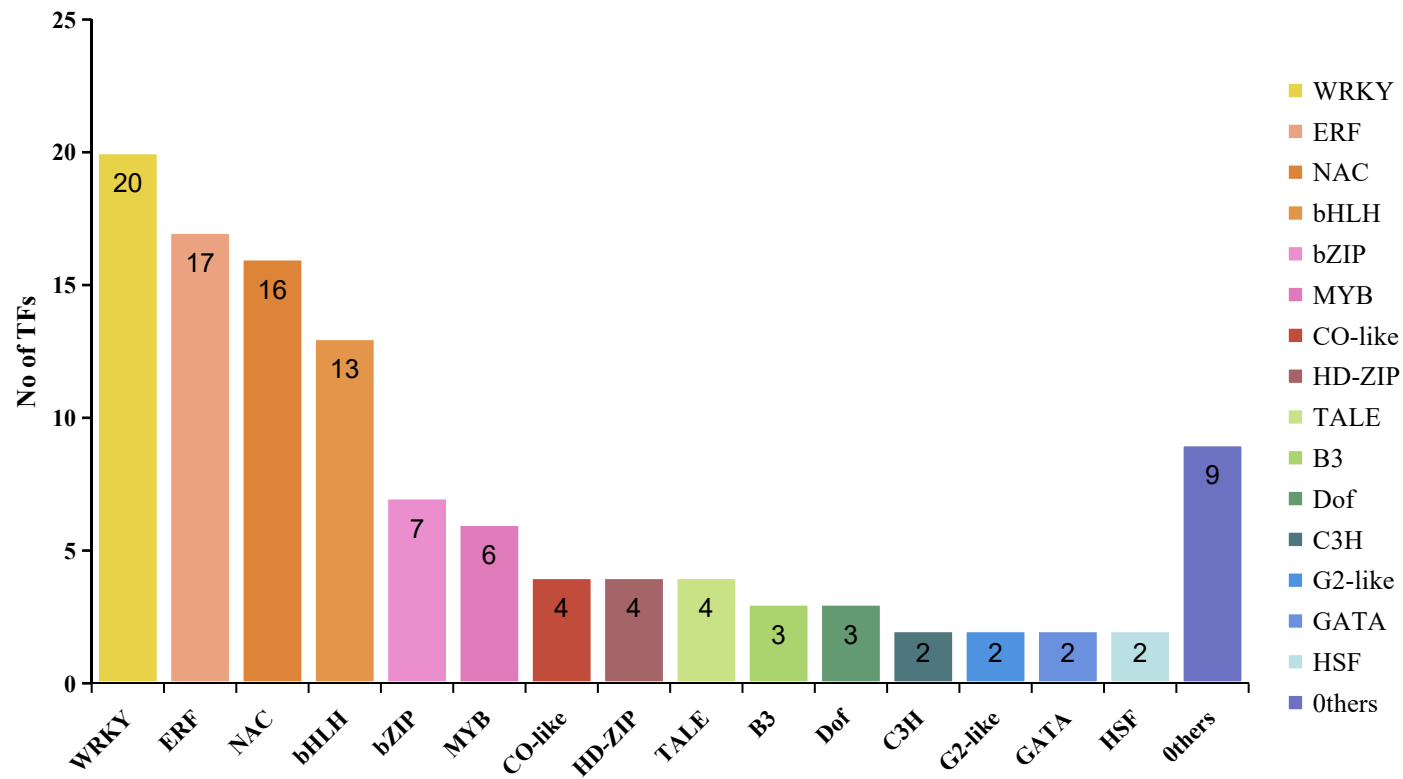

Supplement: Supplementary file 1 [file genes-15-01176-s001.zip › Figure S3. Transcription factors in the salt-tolerant module.pdf]
